# Farm-adapted multicomponent bacteriophage preparation for environmental biocontrol in commercial dairy cattle housing: A One Health approach

**DOI:** 10.14202/vetworld.2026.3883-3898

**Published:** 2026-09-07

**Authors:** Ekaterine Tevdoradze, Nusha Diasamidze, Salome Kalandadze, Nino Tvaradze, Nana Japarashvili, Giorgi Gogoladze, Nato Peradze, Mathias Müsken, Irina Papukashvili, Rati Golijashvili, Svetlana Chubinidze

**Affiliations:** 1 BioChimPharm JSC, Tbilisi, Georgia.; 2 Faculty of Natural Sciences and Medicine, Ilia State University, Tbilisi, Georgia.; 3 National Center for Disease Control and Public Health, R. Lugar Center for Public Health Research, Tbilisi, Georgia.; 4 Swiss Agricultural School Caucasus, Georgia.; 5 Helmholtz Center for Infection Research, Braunschweig, Germany.

**Keywords:** antimicrobial resistance, bacteriophage adaptation, bacteriophage biocontrol, dairy cattle, environmental biocontrol, livestock production, One Health, phage therapy

## Abstract

**Background and Aim:**

The increasing occurrence of antimicrobial-resistant bacteria in livestock environments highlights the need for sustainable alternatives to conventional antimicrobials. Bacteriophages offer a targeted approach for reducing bacterial contamination while supporting antimicrobial stewardship. This study aimed to develop and evaluate a farm-adapted multicomponent bacteriophage preparation for environmental biocontrol in commercial dairy cattle housing.

**Materials and Methods:**

Bacterial strains and bacteriophages were isolated from samples collected at a commercial dairy farm. Selected bacteriophages were characterized for host range, efficiency of plating, multiplicity of infection, and host range expansion through phage adaptation. Purified phage clones were subjected to whole-genome sequencing and bioinformatic analysis. A multicomponent bacteriophage preparation was formulated and lyophilized to facilitate storage and field application. The preparation was subsequently applied to selected environmental surfaces in dairy cattle housing, and bacterial recovery before and after treatment was evaluated using microbiological methods.

**Results:**

Phage screening and adaptation enabled the selection of bacteriophages with complementary activity against bacterial isolates recovered from the dairy farm environment. Adaptation expanded the lytic activity of selected phages against previously less susceptible bacterial hosts. Genomic characterization supported the selection of phages for inclusion in the final preparation, while formulation and lyophilization yielded a preparation suitable for field application. Following environmental application under commercial dairy farm conditions, qualitative changes in bacterial recovery were observed on treated surfaces, demonstrating the feasibility of using a farm-adapted bacteriophage preparation for environmental biocontrol. The findings also demonstrated the practical integration of bacterial surveillance, phage isolation, adaptation, genomic characterization, formulation, and on-farm application within a single biocontrol strategy.

**Conclusion:**

A farm-adapted multicomponent bacteriophage preparation can provide a practical, targeted approach for environmental bacterial control in commercial dairy cattle housing. The combination of locally isolated phages, host range adaptation, genomic characterization, and a field-applicable lyophilized formulation is a key strength of this approach. These findings provide a foundation for further controlled studies using quantitative microbial assessments to determine efficacy, optimize application protocols, and evaluate the contribution of bacteriophage-based environmental interventions to antimicrobial stewardship and One Health strategies in livestock production.

## INTRODUCTION

The One Health approach recognizes the close interconnection among human, animal, and environmental health and promotes integrated, multidisciplinary strategies to address complex health challenges, including antimicrobial resistance (AMR) [1]. Within this framework, monitoring and controlling AMR across interconnected ecosystems have become global priorities for limiting the emergence and dissemination of resistant microorganisms [2, 3]. The widespread use of antibiotics in livestock production has accelerated the emergence and dissemination of AMR, posing substantial threats to animal health, food safety, and public health worldwide. Livestock production accounts for nearly three-quarters of global antimicrobial consumption, with cattle production systems among the major users [4, 5].

In dairy farming, antimicrobials are extensively used to prevent and treat intramammary infections, particularly bovine mastitis, which remains one of the most prevalent and economically important diseases affecting milk quality, productivity, and animal welfare [6–8]. However, repeated and prophylactic antibiotic use can promote the selection of multidrug-resistant (MDR) bacteria and facilitate the horizontal transfer of resistance determinants through manure, milk, wastewater, and environmental runoff [5, 9].

Therefore, sustainable and environmentally compatible alternatives to conventional antimicrobial interventions in livestock production are urgently needed. Among emerging biological approaches, bacterio-phages (phages) have gained renewed scientific attention because of their high host specificity, self-amplifying nature, and ability to selectively eliminate target bacteria while largely preserving commensal microbiota. These characteristics make phages promising candidates for targeted biocontrol, therapeutic applications, and environmental sanitation in veterinary settings [10–12].

Nevertheless, successful field application of phages may be influenced by several factors, including limited activity against mature biofilms, the emergence of phage-resistant bacterial variants, and environmental conditions that affect phage stability. These limitations highlight the need for optimized formulations and application strategies.

In dairy cattle production systems, mastitis-associated pathogens, including *Escherichia coli*, *Staphylococcus aureus*, *Streptococcus uberis*, and *Klebsiella* spp., frequently exhibit resistance to multiple antimicrobial agents [7, 13, 14]. Several studies have demonstrated the potential of phage therapy for controlling mastitis-associated bacteria. Intramammary administration of *E. coli*-specific phage cocktails has been shown to reduce bacterial burden, somatic cell counts, and inflammatory responses [14–16]. Although traditional plaque assays remain the gold standard for evaluating phage activity, recent advances in single-cell technologies offer additional opportunities to investigate phage–host interactions [17–19]. Furthermore, multicomponent phage preparations generally exhibit broader antibacterial activity because their constituent phages have complementary host ranges [19, 20].

Beyond therapeutic applications, phages are increasingly being investigated as environmental biocontrol agents in livestock production systems. Previous studies have demonstrated that phage-based interventions can reduce pathogenic bacteria on animal housing surfaces, equipment, and in wastewater, thereby improving farm hygiene and biosecurity [21–23].

Although phage-based environmental interventions have been investigated in livestock production, their application in dairy cattle housing remains comparatively understudied. Unlike broiler production systems, dairy farms represent long-term housing environments characterized by continuous animal occupancy, persistent moisture, accumulation of organic matter, and repeated environmental exposure, all of which favor pathogen survival and transmission and contribute to diseases such as mastitis [13, 24]. Consequently, evaluating phage-based environmental decontamination in dairy housing addresses an important knowledge gap and extends the potential application of phage biocontrol within livestock production systems [16, 25].

A multicomponent bacteriophage preparation active against seven bacterial genera of major relevance to livestock health (*Escherichia*, *Salmonella*, *Shigella*, *Enterococcus*, *Staphylococcus*, *Pseudomonas*, and *Proteus*) has previously demonstrated efficacy in commercial broiler production systems. However, its application for environmental decontamination in dairy cattle housing has not yet been evaluated. In parallel, stable phage formulations are needed to facilitate storage, transportation, and routine on-farm application under commercial conditions.

Most published studies have focused on poultry or swine production systems, therapeutic applications, or the control of individual bacterial pathogens. Comparatively few studies have evaluated environmental decontamination in commercial dairy housing using multicomponent phage preparations adapted to farm-specific bacterial populations [26, 27]. To our knowledge, few reports have integrated farm-specific bacteriophage isolation, adaptive expansion of the lytic spectrum, lyophilized formulation, and subsequent field application under commercial dairy farm conditions within a single workflow. Within a One Health framework, such an integrated approach could provide a practical model for developing locally adapted phage-based environmental biocontrol strategies that complement conventional farm hygiene practices.

This study aimed to evaluate a farm-adapted multicomponent bacteriophage preparation as an environ-mental biocontrol strategy in commercial dairy cattle housing. Specifically, the objectives were to (1) evaluate the effectiveness of the multicomponent bacteriophage preparation in reducing pathogenic and conditionally pathogenic bacteria under commercial farm conditions; (2) develop and evaluate a lyophilized formulation to improve the practicality, storage stability, and suitability of the preparation for routine on-farm application; and (3) enhance the antibacterial spectrum of the preparation through the isolation of farm-derived bacterial strains, recovery of naturally occurring bacteriophages from farm wastewater, adaptive expansion of the lytic spectrum, and incorporation of a newly isolated and genomically characterized *E. coli* bacteriophage (vB_Eco_BCPEco_001).

Although regulatory frameworks for environmental bacteriophage products continue to evolve in many regions, including the European Union, increasing interest in phage-based alternatives reflects the growing need for sustainable antimicrobial stewardship within One Health strategies.

## MATERIALS AND METHODS

### Ethical approval

This study evaluated the environmental decontamination efficacy of a multicomponent bacteriophage preparation under field conditions and did not involve experimental manipulation of animals. No animals or humans underwent invasive procedures, clinical interventions, sample collection, restraint, or euthanasia. The bacteriophage preparation was applied exclusively to environmental surfaces, including floors, walls, railings, and other housing infrastructure, within a commercial dairy calf facility and was not administered directly to animals.

In accordance with the principles of the Institutional Animal Care and Use Committee and the Public Health Service and U.S. Department of Agriculture Requirements for IACUC Review and Approval (Version 4/20/2012), formal ethical approval was not required because the study did not involve activities requiring institutional animal care and use review or approval. The study was conducted in compliance with applicable national regulations and internationally accepted principles governing environmental biosafety and the agricultural use of bacteriophages, taking into account relevant European Food Safety Authority opinions on bacteriophage safety and the One Health approach.

Permission to conduct the environmental application and access the study site was obtained from the dairy farm owner before commencement of the study. All procedures were performed in accordance with the farm’s biosafety practices and applicable local regulatory requirements.

### Study period and location

The study was conducted between March 13, 2023, and October 10, 2024, at the Georgian-Swiss Agro School “Caucasus” dairy farm in Dmanisi, Georgia. Sampling was performed at multiple time points during this period to characterize bacterial populations circulating within the dairy cattle housing environment and wastewater system. Environmental conditions, including ambient temperature (8–18°C) and relative humidity (65%–75%), were recorded during sampling sessions.

### Study design

The study used a field-based environmental biocontrol design to evaluate a multicomponent veterinary bacteriophage preparation under commercial dairy farm conditions. The experimental workflow comprised isolation and identification of farm-associated bacterial strains, recovery and purification of naturally occurring bacteriophages from farm wastewater, assessment and adaptive expansion of the phage lytic spectrum, characterization of a newly isolated farm-specific *E. coli* bacteriophage (vB_Eco_BCPEco_001), development and stability assessment of a lyophilized formulation, incorporation of the farm-specific phage into the multi-component preparation, and subsequent environmental application under commercial dairy housing conditions.

Environmental samples were collected from defined sites within the dairy facility, and bacterial isolates were characterized to assess susceptibility to the multicomponent phage preparation. Isolates exhibiting low or moderate susceptibility underwent phage adaptation. The optimized preparation was subsequently evaluated under field conditions by comparing bacterial populations before and after environmental application and with those in an untreated control area.

### Bacteriophage preparation

The phage preparation used in this study was a commercially available multicomponent veterinary formulation produced by a Good Manufacturing Practice (GMP)- and ISO 9001:2015-certified biotechnology company (BioChimPharm JSC, Tbilisi, Georgia). The preparation was active against bacteria belonging to seven genera of major veterinary relevance: *Escherichia*, *Salmonella*, *Shigella*, *Enterococcus*, *Staphylococcus*, *Pseudomonas*, and *Proteus*.

Each component consisted of a cocktail of bacteriophages active against multiple serotypes and genotypes of a single bacterial species. The composition of the multicomponent bacteriophage preparation is summarized in Supplementary Table S1. The titer of each individual bacteriophage within a component was 10⁷ plaque-forming units (PFU)/mL. Seven species-specific phage components were combined to prepare the final cocktail, with each individual bacteriophage present at a final concentration of 10⁷ PFU/mL.

The preparation was manufactured exclusively for oral administration in compliance with GMP standards and underwent all required stages of quality control.

To optimize antibacterial activity against farm-specific bacterial populations, a novel lytic *E. coli* bacteriophage, vB_Eco_BCPEco_001, was isolated from wastewater collected at the study farm, genomically characterized, and incorporated into the existing commercial multicomponent bacteriophage preparation. This hybrid formulation combined the broad-spectrum activity of the standardized commercial cocktail with the enhanced specificity of a locally adapted bacteriophage, providing a customized approach to environmental biocontrol under commercial dairy farm conditions.

### Sampling and identification of bacterial strains

Bacterial strains were isolated from various surfaces and materials within the dairy cattle housing environment. Sampling was conducted in multiple stages to monitor the circulating microflora within the facility. To standardize bacterial recovery, sampling was performed over a fixed area of 25 cm² per site, with three independent replicate swabs collected from each sampling point to account for spatial variability. Following collection, all swabs were immediately placed in sterile transport media, maintained on ice at 4°C, and transported to the laboratory within 2 h to preserve microbial viability. The reference strain *E. coli* American Type Culture Collection (ATCC) 12014 was used as a quality-control strain.

A total of 22 samples were collected from seven critical sampling points, including cattle barn floors, stall gratings, cow teats, drinking water reservoirs, feed (hay/straw), and freshly milked cow’s milk. Sampling was performed using wet transport swabs under appropriate biosafety conditions.

Samples were cultured on Tryptic Soy Agar (TSA) and Tryptic Soy Broth (TSB) (Biolife Italiana srl., Milan, Italy; Lot No. 402150-SG2309) and incubated at 37°C for 16–18 h. Samples from each sampling site were processed in triplicate to improve the reliability of bacterial recovery. Following primary cultivation, bacterial isolates were subcultured onto appropriate selective media for qualitative isolation and presumptive identification based on colony morphology and conventional microbiological characteristics.

Species-level identification was confirmed using the VITEK® MS PRIME matrix-assisted laser desorption/ionization time-of-flight mass spectrometry system (MALDI-TOF MS; score ≥2.0) and VITEK® 2 Compact system (bioMérieux, Marcy-l’Étoile, France). Selective media and species-level identification methods are summarized in Supplementary Table S2. Identified isolates were cultured in TSB at 37°C and stored at −80°C in 30% glycerol until further analysis.

### Phage isolation and purification

Novel bacteriophages were isolated from sewage samples collected from the wastewater system of the dairy cattle farm. A total of six independent sewage samples were collected at different time points during the study period to capture temporal variation in the circulating bacterial population within the farm wastewater system.

Farm-derived *E. coli* isolates were used as host bacteria and cultured in TSB (Biolife Italiana srl., 402150-SG2309). Samples were centrifuged at 6,000 × *g* for 10 min at 4°C and filtered through 0.45-µm membrane filters. For enrichment, 1 mL of exponentially growing host culture [optical density at 600 nm (OD₆₀₀) = 0.6; approximately 10⁸ colony-forming units (CFU)/mL] was added to 90 mL of sewage filtrate supplemented with 10 mL of 10× TSB and incubated at 37°C for 24 h with shaking at 180 rpm [28]. Negative controls consisting of sewage filtrate processed in parallel without host bacteria were included to exclude environmental contamination or spontaneous phage induction. No plaque formation was observed in the negative controls throughout the enrichment procedure. All enrichment experiments were performed in triplicate.

Following enrichment, lysates were centrifuged at 12,000 × *g* for 10 min and filtered through 0.22-µm membrane filters. Phage activity was screened using the double-layer agar overlay method, with bottom agar containing 1.5% (w/v) agar and top agar containing 0.7% (w/v) agar supplemented with host bacteria (approximately 10⁸ CFU/mL). Plates were incubated at 37°C for 16–18 h [17, 29]. Clear plaques were considered indicative of lytic phage activity.

Spot assays were performed by overlaying host bacteria onto agar plates and applying 10–20 µL of filtered lysate onto the bacterial lawn [30, 31]. All plaque assays and spot tests were conducted in triplicate.

Individual plaques were picked using sterile tips and suspended in SM buffer prepared in-house in Tbilisi, Georgia using reagents purchased from Sigma-Aldrich (St. Louis, MO, USA). The buffer contained 50 mM Tris-HCl (pH 7.5), 100 mM NaCl, 8 mM MgSO₄, and 0.01% gelatin. Plaques were subjected to four to six successive rounds of purification using the double-layer agar method to ensure clonal homogeneity. Each purification step was performed in triplicate to confirm reproducibility.

For propagation, the purified phage was amplified in mid-log-phase *E. coli* cultures (OD₆₀₀ = 0.4–0.6) at a multiplicity of infection (MOI) of 0.01–0.1 until complete lysis. Lysates were clarified and filtered as described above. Scale-up was performed to a final volume of 500 mL–1 L, yielding titers ≥10¹⁰ PFU/mL. Propagation experiments were conducted in at least two independent replicates. The purified phage, designated vB_Eco_BCPEco_001, was stored in SM buffer at 4°C until use.

### Lytic spectrum and host range analysis

The lytic activity of the multicomponent phage preparation and the newly isolated *Escherichia* phage was evaluated against target bacterial strains. The preparation was tested against 38 bacterial strains isolated from the dairy cattle facility using the spot-test method [28, 30]. Briefly, 100 µL of logarithmic-phase bacterial culture (10⁹ CFU/mL) was mixed with 4–5 mL of semisolid Tryptic Soy Agar (TSA; 0.7% w/v) and poured onto TSA (1.5%) plates. After solidification, 10–20 µL of the phage preparation (10⁷ PFU/mL) was spotted onto the bacterial lawn (10⁸ CFU/mL). Plates were incubated overnight at 37°C. Clear spots indicated phage activity, and the time required for bacterial clearance was used to assess phage lytic activity [18, 30, 31].

Lysis patterns were recorded using the following scale: 4+ = confluent lysis; 3+ = semi-confluent lysis; 2+ = less than semi-confluent lysis; 1+ = opaque lysis; ± = less than opaque lysis; and −/R = no lysis or resistance.

The *Escherichia* phage vB_Eco_BCPEco_001 was evaluated against 10 *E. coli* strains. Based on the spot-test results, plaque formation and efficiency of plating (EOP) were assessed using the double-layer agar overlay method. EOP was calculated as the ratio of PFU obtained on the test bacterium to those obtained on the reference host bacterium, *E. coli* ATCC 12014. For each assay, exponentially growing bacterial cultures (10⁸ CFU/mL) were mixed with phage lysate (10⁷ PFU/mL) at an MOI of 0.01. All experiments were performed in triplicate. EOP values were categorized as high (≥0.5), medium (0.1–0.5), low (0.001–0.1), or no lysis (≤0.001).

Adaptation of the multicomponent phage preparation was performed individually for each bacterial isolate exhibiting low or moderate susceptibility to the preparation using a modified Appelmans technique, a well-established method for directed bacteriophage adaptation and expansion of host range [32]. Briefly, 10-fold serial dilutions of the phage cocktail were prepared while maintaining a constant bacterial concentration. After incubation, the lysate from the lowest phage concentration that produced complete bacterial lysis was filtered through a 0.22-µm membrane filter and used for the subsequent adaptation cycle with the same bacterial isolate. The procedure was repeated until no further reduction in the minimum effective phage concentration required for complete lysis was observed, indicating completion of the adaptation process. The number of adaptation cycles varied among bacterial isolates and depended on the point at which no further improvement in lytic activity was achieved. A detailed step-by-step description of the phage adaptation procedure is provided in Supplementary Method S3, whereas the lytic activity of all 38 bacterial isolates before and after adaptation is summarized in Table 1.

### Transmission electron microscopy (TEM)

High-purity phage preparations were examined using TEM. Phage samples were adsorbed onto carbon-coated mica, washed, and negatively stained with 2% aqueous uranyl acetate (pH 5.0). Samples were examined using a TEM 910 transmission electron microscope (Carl Zeiss AG, Oberkochen, Germany) at 80 kV. Images were recorded using a slow-scan charge-coupled device camera and analyzed to determine phage morphology. Ten phage particles were imaged (n = 10), and particle dimensions were determined using ITEM software version 5.2 (build 3554; Olympus Soft Imaging Solutions, Münster, Germany).

### Lyophilization and stability assessment

Phage suspensions were lyophilized in the presence of 1.4%–1.6% (w/v) maltodextrin as an excipient and lyoprotectant. One liter of *E. coli* monophage vB_Eco_BCPEco_001 suspension (10¹⁰ PFU/mL) was subjected to a two-stage freeze-drying cycle. Primary drying was performed at −40°C for 12 h under a vacuum of 0.13 mbar to facilitate sublimation of ice from the frozen matrix. Secondary drying was performed by gradually increasing the temperature from −40°C to 36°C over 36 h to remove residual bound moisture [33–35]. The resulting lyophilized phage preparation was sealed and stored under dry conditions. Lyophilization was performed using a LYOPRO-1-CIP freeze dryer (LYOMAC Lyo-23-005 V4.0).

To assess stability, lyophilized *E. coli* monophage preparations were stored at 4°C and 25°C for up to 6 months, with the initial pre-lyophilization phage suspension serving as the control. Stability was evaluated after 1, 3, and 6 months of storage. Samples were reconstituted in sterile 0.9% NaCl (1 g/mL), serially diluted, and viable phage titers were determined using the double-agar overlay assay. PFU were quantified as an indicator of viable phage particles.

The newly isolated farm-specific *E. coli* phage vB_Eco_BCPEco_001 was stabilized by maltodextrin-based lyophilization before incorporation into the multicomponent veterinary bacteriophage preparation. This approach enabled the integration of a genomically characterized, locally adapted phage into an existing commercial formulation, providing a practical strategy for customizing phage preparations intended for environmental application under commercial dairy farm conditions.

### Environmental application on the dairy cattle farm

The study was conducted under routine management conditions at a commercial dairy farm housing approximately 70–80 dairy cattle to evaluate the practical feasibility of integrating phage-based environmental decontamination into routine farm biosecurity programs. Calves were maintained in a group-housing system on concrete flooring with straw bedding. The calf housing area was mechanically cleaned once daily according to the farm’s standard hygiene protocol, and no chemical disinfectants were used during the experimental period. Identical management and husbandry practices were maintained in the experimental and control areas throughout the study. The phage preparation was applied once daily for five consecutive days, and environmental sampling was performed before treatment and 2 weeks after completion of the application protocol to assess qualitative changes in the environmental bacterial community under commercial field conditions.

Two physically separated calf housing areas, each measuring 50 m², were designated as the experimental and control areas. Each area housed six calves at a stocking density of approximately 8.3 m²/animal. The two areas were located approximately 100 m apart and were managed independently throughout the study. To minimize the risk of cross-contamination, dedicated personnel and equipment were assigned to each area during sampling and phage application. Phage spraying was performed exclusively within the experimental area under low-airflow conditions, and no cleaning equipment was shared between the two areas during the study period. To ensure comparability and minimize potential confounding factors, environmental parameters, including temperature (8–18°C) and relative humidity (60%–70%), were monitored daily in both areas throughout the study period.

Phage treatment was performed using a seven-component commercial bacteriophage preparation at a final concentration of 10⁷ PFU/mL for each component, combined with the farm-specific *E. coli* monophage. The farm-specific phage was supplied as a lyophilized preparation (100 g; stock titer = 10⁹ PFU/g) and reconstituted immediately before application in 10 L of sterile 0.9% NaCl under continuous stirring at room temperature (22°C) for 5–10 min until complete dissolution, yielding a working concentration of 10⁷ PFU/mL. The reconstituted suspension was used immediately after preparation.

The preparation was applied once daily for five consecutive days by spraying at 200 mL/m², corresponding to a phage dose of 2 × 10⁹ PFU/m² per application and a cumulative dose of 1 × 10¹⁰ PFU/m². Application was performed using a STAR 16 AGRO manual backpack sprayer (Ref. 83951) equipped with a metallic adjustable conical nozzle and operated at 1.5 bar with a flow rate of 0.7 L/min, according to the manufacturer’s specifications. To ensure uniform coverage, the phage suspension was applied at a constant rate of 200 mL/m² using standardized spraying conditions throughout the treated area.

The control area received no phage treatment. To assess safety, a licensed veterinarian conducted daily clinical examinations of all calves, focusing on signs of respiratory distress, enteric illness, and behavioral changes. No adverse effects, illness, or stress-related symptoms were observed during or after application.

Environmental sampling was repeated 2 weeks after completion of treatment to evaluate changes in the bacterial community following phage application. Samples from each sampling site were collected in triplicate to improve the reliability of bacterial recovery. Bacterial isolates were cultured on appropriate selective media for qualitative isolation and species-level identification (Supplementary Table S2). Treatment efficacy was assessed by comparing bacterial species composition before and after phage application in the experimental and control areas.

### Genome sequencing and bioinformatic analysis

DNA extraction was performed using the Qiagen DNA Extraction and Purification Kit. Library preparation was performed using the Illumina DNA Prep Kit. (Illumina Inc., San Diego, CA, USA) Library quality control included quantification using a Qubit fluorometer (Thermo Fisher Scientific Inc., Waltham, MA, USA) and fragment-size estimation using the TapeStation 4200 system. Sequencing was performed using the Illumina MiSeq Dx platform with the 500-cycle v2 kit.

Read quality-control, trimming, and *de novo* assembly were performed using CLC Genomics Workbench version 24.0.1 (Qiagen). The trimming quality limit was set at 0.025. The complete phage genome was obtained at a read-depth coverage of 150×. The assembled genome was annotated using Pharokka [36]. The presence of virulence and antibiotic resistance genes was assessed through gene-family analysis using EDGE Bioinformatics (Los Alamos National Laboratory, Los Alamos, NM, USA).

Similar phage genomes were identified in the National Center for Biotechnology Information (NCBI) nucleotide database using Nucleotide Collection (nr/nt) Basic Local Alignment Search Tool (BLAST). kSNP4 [37] was used to detect single-nucleotide polymorphisms (SNPs) across the phage genomes. A k-mer size of 19 was calculated using Kchooser4. MrBayes was used for phylogenetic tree reconstruction [38], and FigTree was used for tree visualization. Intergenomic similarities between vB_Eco_BCPEco_001 and reference strains retrieved from NCBI were calculated using the Virus Intergenomic Distance Calculator (VIRIDIC) [39]. The VIRIDIC BLASTN parameters were word_size = 7, reward = 2, penalty = −3, gapopen = 5, and gapextend = 2. The genome sequence of *Escherichia* phage vB_Eco_BCPEco_001 is available in GenBank under accession number PV295200.

### Statistical analysis

One-way analysis of variance (ANOVA) was used to compare EOP values across multiple host groups. Assays were performed in triplicate, and the results are presented as the mean ± standard deviation of three independent experiments. Stability experiments were conducted using two independent biological replicates. The effects of storage temperature and time on phage viability were analyzed using two-way ANOVA followed by Tukey’s post hoc multiple-comparison test.

A p < 0.05 was considered statistically significant. All statistical analyses were performed using Minitab Statistical Software version 16.2.4.

## RESULTS

### Bacterial strains

Bacteriological analysis of 22 environmental samples yielded approximately 84 pure bacterial cultures. MALDI-TOF MS enabled species-level identification of 62 isolates. After excluding saprophytic bacteria, 38 pathogenic and conditionally pathogenic bacterial strains were retained for further analysis, including *E. coli* (n = 10), *Enterococcus faecium* (n = 4), *Enterococcus faecalis* (n = 2), *Enterococcus mundtii* (n = 1), *Enterobacter cloacae* (n = 1), *Enterococcus durans* (n = 1), *Pantoea* spp. (n = 1), *Proteus vulgaris* (n = 2), *Staphylococcus haemolyticus* (n = 4), *Staphylococcus hominis* (n = 3), *Staphylococcus sciuri* (n = 2), *Staphylococcus chromogenes* (n = 1), *Staphylococcus xylosus* (n = 2), *Staphylococcus equorum* (n = 1), *Staphylococcus warneri* (n = 1), and *P. aeruginosa* (n = 2) (Table 1).

### Isolation and morphology of *Escherichia* phage vB_Eco_BCPEco_001

A novel *Escherichia* bacteriophage was isolated from sewage collected at the Georgian-Swiss Agro School “Caucasus” dairy cattle farm. Ten farm-derived *E. coli* strains and the reference strain *E. coli* ATCC 12014 were used for phage isolation. A single lytic phage was recovered using strains N20/2v, 010/1F, and N23/2v as hosts. The phage formed clear plaques approximately 2 mm in diameter and was designated vB_Eco_BCPEco_001 (Figure 1). TEM revealed an icosahedral head and a contractile tail, consistent with the morphological characteristics of the family *Myoviridae*. The mean particle dimensions were as follows: head width, 92 ± 5 nm; head length, 117 ± 7 nm; tail length, 112 ± 5 nm; and overall length, 229 ± 8 nm.

**Figure 1:**
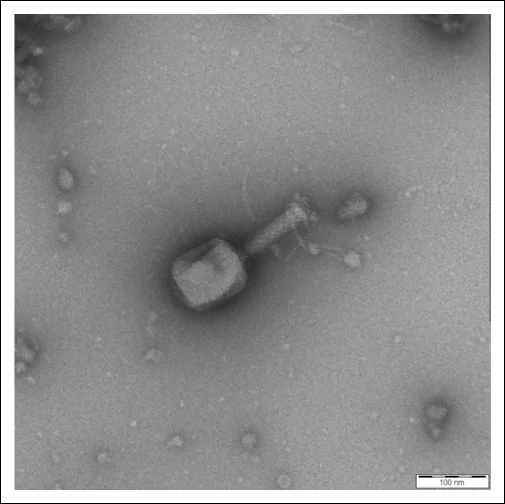
Transmission electron micrograph of *Escherichia coli* bacteriophage vB_Eco_BCPEco_001 obtained by negative staining. The phage displays an icosahedral head and a contractile tail, consistent with members of the family *Myoviridae*. Scale bar = 100 nm.

### Lytic spectrum of the phage preparation and host range analysis

Thirty-eight bacterial strains were screened for susceptibility using spot-test assays. The multicomponent bacteriophage preparation demonstrated lytic activity against bacterial species belonging to *Escherichia*, *Salmonella*, *Shigella*, *Proteus*, *Staphylococcus*, *Pseudomonas*, and *Enterococcus* (Table 1). Lysis was scored using a semiquantitative scale ranging from confluent lysis (4+) to resistance (−/R).

Initial testing showed that 42% (16/38) of isolates exhibited high susceptibility, defined as a lysis score of 3+ or 4+. Serial phage adaptation markedly enhanced lytic activity in isolates with initially lower susceptibility. Following adaptation, all 38 isolates exhibited high susceptibility, with 12 isolates showing a score of 3+ and 26 showing a score of 4+ (Table 1).

The isolated phage vB_Eco_BCPEco_001 displayed strong lytic activity against 80% of the tested *E. coli* strains. EOP analysis demonstrated productive infection, with EOP values ≥0.1 for most susceptible strains (Table 2), supporting the inclusion of this phage as an additional component of the preparation.

Incorporating the newly isolated phage, along with adaptive host range expansion, increased high-susceptibility coverage from 42% before adaptation to all 38 tested isolates after optimization.

**Table 1:** Lytic activity of the multicomponent bacteriophage preparation against pathogenic and conditionally pathogenic bacterial strains isolated from a dairy cattle environment.

**Source**	**Strain ID**	**Bacterial strain**	**Before adaptation**	**After adaptation**
Stall grating	01/1F	Enterococcus faecium	1+ (OL)	4+ (CL)
	02/1F	Escherichia coli	1+ (OL)	4+ (CL)
	015/1F	Staphylococcus haemolyticus	4+ (CL)	4+ (CL)
	016/1F	Pseudomonas aeruginosa	3+ (SCL)	4+ (CL)
	N1/2v	E. coli	2+ (<SCL)	3+ (SCL)
	N3/2v	Enterobacter cloacae	1+ (OL)	4+ (CL)
	N4/2v	E. coli	± (<OL)	4+ (CL)
	N6/2v	E. coli	1+ (OL)	3+ (SCL)
	N18/2v	Enterococcus faecalis	2+ (<SCL)	4+ (CL)
	N20/2v	E. coli	1+ (OL)	3+ (SCL)
Cattle barn floor	09/1F	Staphylococcus chromogenes	4+ (CL)	4+ (CL)
	010/1F	E. coli	± (<OL)	3+ (SCL)
	017/1F	Staphylococcus xylosus	3+ (SCL)	4+ (CL)
	021/1F	Staphylococcus haemolyticus	4+ (CL)	4+ (CL)
	022/1F	S. haemolyticus	4+ (CL)	4+ (CL)
	02/2F	E. coli	3+ (SCL)	4+ (CL)
	06/2F	E. faecium	1+ (OL)	3+ (SCL)
	07/2F	*Proteus vulgaris*	1+ (OL)	3+ (SCL)
	017/2F	Staphylococcus hominis	3+ (SCL)	4+ (CL)
	022/2F	Staphylococcus equorum	4+ (CL)	4+ (CL)
	N13/2v	E. faecium	2+ (<SCL)	4+ (CL)
	N19/2v	P. aeruginosa	3+ (SCL)	4+ (CL)
	N23/2v	E. coli	1+ (OL)	3+ (SCL)
Feed (hay/straw)	01/2F	Enterococcus durans	1+ (OL)	3+ (SCL)
	04/2F	P. vulgaris	2+ (<SCL)	3+ (SCL)
	03/2F	*Staphylococcus * *sciuri*	2+ (<SCL)	4+ (CL)
	08/2F	*Staphylococcus * *xylosus*	4+ (CL)	4+ (CL)
	09/2F	Staphylococcus warneri	4+ (CL)	4+ (CL)
	013/2F	E. faecium	2+ (<SCL)	3+ (SCL)
	014/2F	S. haemolyticus	2+ (<SCL)	3+ (SCL)
	015/2F	E. coli	3+ (SCL)	4+ (CL)
	023/2F	S. hominis	3+ (SCL)	4+ (CL)
	024/2F	S. hominis	3+ (SCL)	4+ (CL)
	N9/2v	Enterococcus mundtii	± (<OL)	4+ (CL)
	N10/2v	Pantoea spp. (genus Enterobacter)	4+ (CL)	4+ (CL)
	N12/2v	E. coli	± (<OL)	3+ (SCL)
	N24/2v	E. faecalis	2+ (<SCL)	4+ (CL)
	N25/2v	S. sciuri	2+ (<SCL)	4+ (CL)

Lysis score values: CL = confluent lysis; SCL = semi-confluent lysis; OL = opaque lysis; 4+ = CL; 3+ = SCL; 2+ = less than SCL; 1+ = OL; ± = less than OL; −/R = no lysis/resistant.

**Table 2:** Host range and efficiency of plating of Escherichia phage vB_Eco_BCPEco_001.

**Source**	**E. coli** ** strain ID**	**Spot-test**	**High EOP (≥0.5)**	**Moderate EOP (0.1–0.5)**	**Low EOP (≤0.1)**
Stall grating	02/1F	2+ (<SCL)	−	0.12	−
	N1/2v	2+ (<SCL)	−	0.2	−
	N4/2v	2+ (<SCL)	0.6	–	−
	N6/2v	1+ (OL)	−	−	0.002
	N20/2v	3+ (SCL)	0.7	−	−
Cattle barn floor	010/1F	4+ (CL)	1	−	−
	02/2F	2+ (<SCL)	−	0.16	−
	N23/2v	3+ (SCL)	1.4	−	−
Feed (hay/straw)	015/2F	2+ (<SCL)	−	−	0.08
	N12/2v	3+ (SCL)	0.6	−	−
Reference	ATCC 12014	4+ (CL)	1	−	−

CL = confluent lysis; EOP = efficiency of plating; OL = opaque lysis; SCL = semi-confluent lysis. Lysis patterns: 4+ = CL; 3+ = SCL; 2+ = less than SCL; 1+ = OL; ± = less than OL; −/R = no lysis/resistant. EOP categories: high = ≥0.5; medium = 0.1–0.5; low = 0.001–0.1; no lysis = ≤0.001.

### Stability of the lyophilized phage preparation

Following lyophilization and reconstitution, bacteriophage vB_Eco_BCPEco_001 maintained high viability. Phage titers decreased only slightly, from 1 × 10¹⁰ PFU/mL before lyophilization to 5 × 10⁹–9 × 10⁹ PFU/mL after reconstitution, corresponding to a reduction of <0.3 log₁₀ PFU/mL.

Two-way ANOVA showed no statistically significant differences in phage titers between storage temperatures (4°C and 25°C) or among storage periods (1, 3, and 6 months) (p > 0.05). Comparable phage titers were maintained throughout the study under both storage conditions.

These results demonstrated that the lyophilized formulation maintained phage stability and infectivity for up to 6 months under the evaluated storage conditions.

### Environmental application on the dairy cattle farm

Before treatment, bacteriological analysis of cattle housing surfaces revealed diverse saprophytic and opportunistic pathogenic microbiota (Table 3). The phage preparation was applied to the experimental area once daily for five consecutive days.

Post-treatment analysis showed a reduced diversity of opportunistic pathogenic bacteria recovered from the treated environmental sites compared with pretreatment findings. *E. coli* was no longer recovered from treated feed samples, whereas *P. aeruginosa*, *S. sciuri*, and *P. vulgaris* were no longer recovered from treated cattle barn floor samples. In contrast, the untreated control area retained bacterial profiles more similar to those observed before treatment (Table 3).

Throughout the trial, daily veterinary clinical examinations identified no adverse effects associated with phage application. Calves in the experimental group maintained normal behavior and appetite and showed no signs of respiratory or enteric illness during or after treatment.

The environmental application targeted a polymicrobial community comprising several opportunistic bacterial genera, including *Escherichia*, *Staphylococcus*, *Pseudomonas*, and *Enterococcus*, under commercial dairy housing conditions characterized by continuous animal occupancy and organic matter accumulation.

**Table 3:** Changes in bacterial populations following application of the multicomponent veterinary bacteriophage preparation in a dairy cattle housing environment.

**Source**	**Before phage treatment: experimental area**	**After phage treatment: experimental area**	**No phage treatment: control area**
Stall grating	Bacillus altitudinis/Bacillus pumilus; Bacillus subtilis/Bacillus amyloliquefaciens/Bacillus vallismortis; S. sciuri; S. haemolyticus; Streptococcus equinus; S. chromogenes; Serratia rubidaea; P. aeruginosa	B. altitudinis/B. pumilus; B. subtilis/B. amyloliquefaciens/B. vallismortis; Paenibacillus pabuli; S. rubidaea	B. altitudinis/B. pumilus; B. subtilis/ B. amyloliquefaciens/B. vallismortis; S. sciuri; S. haemolyticus; S. equinus; S. chromogenes; S. rubidaea; P. aeruginosa; P. pabuli
Feed (hay/straw)	B. subtilis/B. amyloliquefaciens/B. vallismortis; S. equinus; Staphylococcus caprae; Staphylococcus gallinarum; E. cloacae/Enterobacter asburiae; E. coli	B. subtilis/B. amyloliquefaciens/B. vallismortis; E. mundtii	B. subtilis/B. amyloliquefaciens/ B. vallismortis; S. equinus; Citrobacter freundii; S. gallinarum; E. cloacae/ E. asburiae; Macrococcus caseolyticus
Cattle barn floor	P. aeruginosa; S. sciuri; Corynebacterium urealyticum; S. rubidaea; P. vulgaris	C. urealyticum; S. rubidaea	P. aeruginosa; S. sciuri; C. urealyticum; S. rubidaea; P. vulgaris

### Comparative genomic analysis of vB_Eco_BCPEco_001

The complete genome of *Escherichia* phage vB_Eco_BCPEco_001 was obtained by *de novo* assembly and consisted of 170,117 bp of linear double-stranded DNA with a guanine–cytosine content of 35.2%. A total of 295 genes were identified, including 287 coding sequences and eight transfer RNA genes. No virulence- or antibiotic resistance-associated genes were detected, and no integrase-coding sequence was identified. Genome assembly and annotation statistics are summarized in Table 4. The genome sequence is available in NCBI under accession number PV295200.

**Table 4:** Genome assembly and annotation statistics of Escherichia phage vB_Eco_BCPEco_001.

**Phage**	**Number of contigs**	**Total length (bp)**	**Genes**	**Coding sequences**	**tRNA genes**	**Guanine–cytosine content (%)**	**Read coverage (×)**
vB_Eco_BCPEco_001	1	170,117	295	287	8	35.2	150

Phylogenetic analysis based on whole-genome SNPs showed that vB_Eco_BCPEco_001 clustered closely with phages YUEEL01, OES_C-1, and AV121, with a posterior probability of 0.87 (Figure 2).

**Figure 2:**
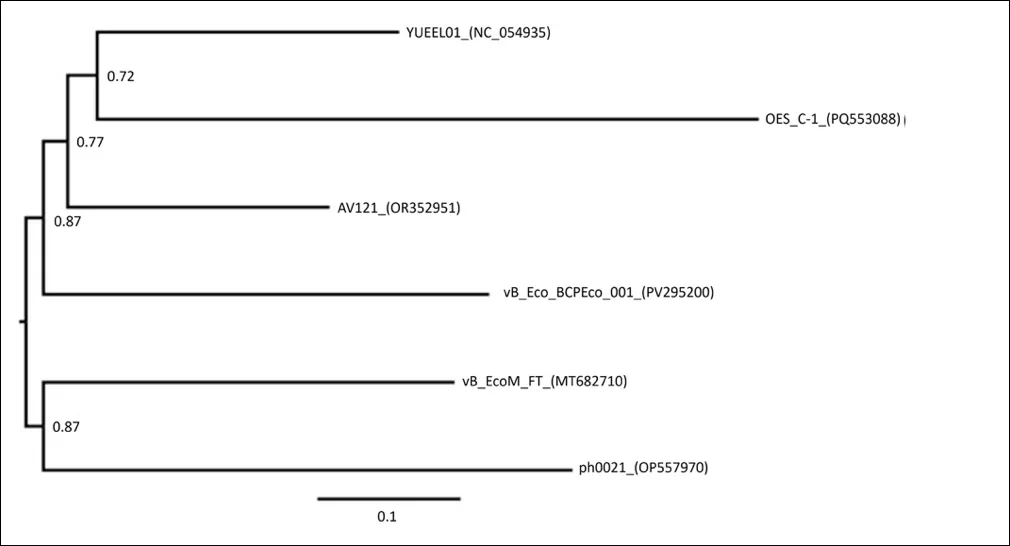
Rooted Bayesian phylogenetic tree of *Escherichia coli* bacteriophages based on whole-genome sequences. Posterior probability values are shown at the branch nodes. The scale bar represents substitutions per site.

Intergenomic similarity analysis using VIRIDIC revealed similarity values of 89.6%–92.9% between vB_Eco_BCPEco_001 and closely related phages, consistent with classification within the same genus according to International Committee on Taxonomy of Viruses (ICTV) criteria (Figure 3). More distant relationships were observed with phages vB_EcoM_FT and ph0021.

**Figure 3:**
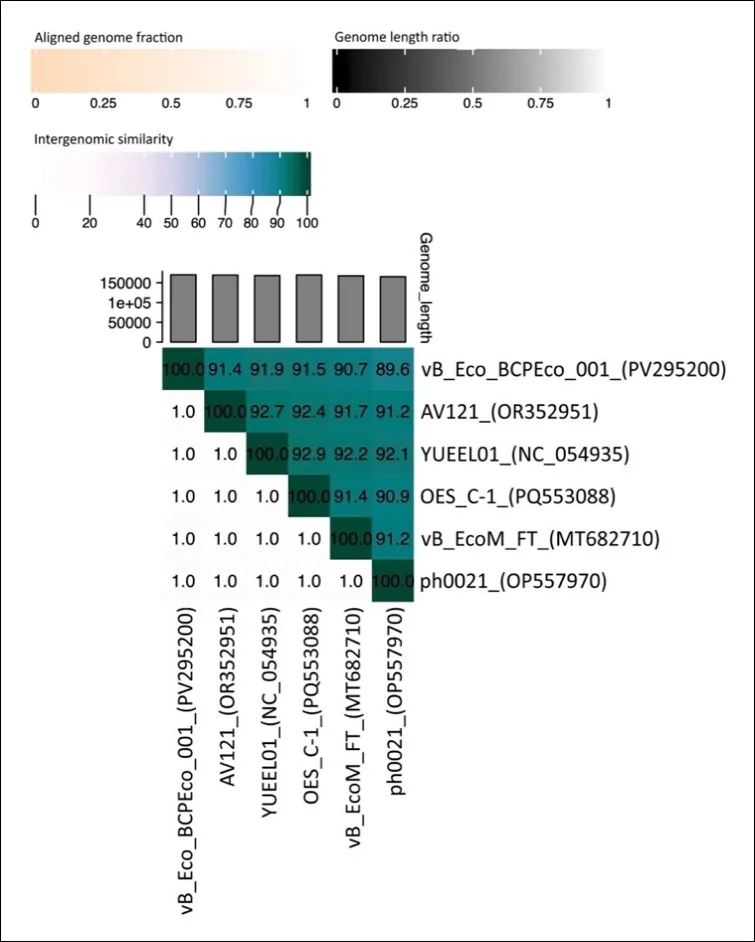
VIRIDIC heatmap showing intergenomic similarities between *Escherichia coli* phage vB_Eco_BCPEco_001 and related bacteriophages.

## DISCUSSION

### Environmental bacterial contamination and relevance to One Health

This study evaluated the feasibility of applying a multicomponent bacteriophage preparation as an environmental biocontrol agent in a commercial dairy cattle farm. Bacteriological analyses revealed diverse microbiota on cattle housing surfaces, including several pathogenic and conditionally pathogenic bacterial species commonly associated with bovine infections and environmental contamination. These findings are consistent with previous reports identifying livestock housing environments as important reservoirs of opportunistic pathogens that may contribute to infection pressure within production systems [14, 21, 25]. From a One Health perspective, these environments are important interfaces where animal, environmental, and potential human health risks converge, emphasizing the need for effective and sustainable microbial control strategies [2, 5, 22, 40].

Unlike previous studies that primarily examined phage-based decontamination of dairy equipment or wastewater using liquid preparations under controlled conditions [24, 41], the present study evaluated a multicomponent phage preparation applied directly to dairy cattle housing surfaces under commercial farm conditions. The study also incorporated isolation of farm-specific bacteria and bacteriophages, genomic characterization of the novel lytic *E. coli* phage vB_Eco_BCPEco_001, and expansion of the preparation’s lytic spectrum through adaptive serial passaging.

### Adaptive expansion of the bacteriophage lytic spectrum

Initial susceptibility testing showed that 42% of the farm-derived bacterial isolates were highly susceptible to the multicomponent phage preparation before adaptation. Serial phage passaging substantially broadened the lytic spectrum, resulting in high susceptibility among all tested isolates after adaptation. This finding is consistent with previous studies demonstrating that phage cocktails can be optimized through adaptive processes to improve their efficacy against heterogeneous bacterial populations [19, 23]. Furthermore, high-resolution analyses of lytic kinetics, as proposed in recent methodological studies [18], could provide greater precision for monitoring adaptive changes in phage–host range.

The capacity to adapt bacteriophages to locally circulating bacterial strains may represent an important advantage of phage-based interventions, particularly in environments characterized by heterogeneous bacterial populations. Such adaptability may also be relevant to One Health-oriented strategies intended to reduce reliance on routine antimicrobial and biocide use [11].

### Farm-specific *Escherichia* phage and genomic characteristics

Isolation and incorporation of the novel *E. coli* phage vB_Eco_BCPEco_001 further expanded the antibacterial coverage of the preparation. The phage demonstrated lytic activity against the majority of tested *E. coli* isolates, and EOP analysis confirmed productive infection in susceptible strains. These findings supported its incorporation as an additional farm-specific component of the multicomponent preparation.

Whole-genome analysis identified no detectable virulence- or AMR-associated genes and no integrase-coding sequence. These genomic characteristics are important considerations when selecting bacteriophages intended for veterinary or environmental applications and are consistent with current biosafety considerations for bacteriophage-based products [42]. The genomic characterization conducted in this study therefore provided additional information supporting the selection of vB_Eco_BCPEco_001 for incorporation into the preparation.

### Lyophilization and formulation stability

From a formulation perspective, lyophilization using maltodextrin as a lyoprotectant resulted in minimal loss of phage viability, with titers remaining close to pre-lyophilization values after reconstitution. In addition, no significant differences in phage titers were detected between the evaluated storage temperatures or among storage periods of up to 6 months.

Previous studies have primarily investigated the lyophilization of individual bacteriophages intended for therapeutic or experimental applications [33, 35]. In contrast, the present study demonstrated the practical incorporation of a lyophilized farm-specific phage into a multicomponent preparation intended for environmental biocontrol under commercial dairy farm conditions [34]. Maintaining phage viability during storage and subsequently incorporating the reconstituted preparation into an on-farm application protocol may improve the practicality of phage-based approaches for routine biosecurity programs.

### Environmental application under commercial farm conditions

Field application of the phage preparation resulted in a qualitative shift in the bacterial species recovered from treated environmental sites. Following treatment, *E. coli* was no longer recovered from treated feed samples, whereas *P. aeruginosa*, *S. sciuri*, and *P. vulgaris* were no longer recovered from treated cattle barn floor samples. In contrast, the untreated control area retained a bacterial profile more similar to baseline conditions. These observations suggest that environmental phage application can alter the composition of recoverable bacterial populations under commercial dairy farm conditions.

Importantly, the preparation was applied to environmental surfaces rather than directly to animals. Daily veterinary examinations identified no observable adverse effects associated with the treatment, and the calves maintained normal behavior and appetite without signs of respiratory or enteric illness during the observation period. Thus, under the conditions evaluated in this study, environmental application of the bacteriophage preparation was compatible with routine calf housing and management practices.

By reducing the recovery of selected opportunistic pathogenic bacteria from environmental surfaces, phage-based environmental interventions may support strategies to limit microbial circulation within the animal–environment interface. This concept is consistent with the broader One Health framework, which emphasizes interconnected approaches to animal, environmental, and human health [1, 40].

Conducting the study under routine commercial farm conditions increases the practical relevance of the findings because the intervention was evaluated in an environment characterized by continuous animal occupancy, organic matter accumulation, and routine husbandry practices. Nevertheless, larger multicenter studies with longer observation periods are required to determine whether these effects are reproducible across different dairy production systems and environmental conditions.

By integrating farm-specific bacteriophage isolation, adaptive host range expansion, lyophilization, genomic characterization, and field application within a single workflow, the present study extends previous approaches to environmental phage biocontrol in commercial dairy housing. The findings demonstrate the feasibility of incorporating a farm-adapted bacteriophage preparation into routine dairy farm biosecurity practices and provide a basis for further validation under diverse commercial farming conditions.

### Limitations and future research directions

This study has several limitations. First, it was designed to evaluate qualitative changes in the environmental bacterial community through species-level identification rather than quantitative enumeration of bacterial loads. Environmental sampling was performed only before treatment and 2 weeks after completion of phage application, and phage persistence on treated surfaces, expressed as PFU/cm², was not evaluated. Consequently, the magnitude and temporal dynamics of bacterial reduction and environmental phage persistence could not be determined. Future studies should incorporate sequential sampling, quantitative microbiological analyses, and appropriate statistical evaluation to characterize treatment kinetics and environmental persistence more precisely.

Second, no phage-neutralizing agent was used during post-treatment sampling. Because the primary objective was qualitative characterization of recovered bacterial species rather than quantitative determination of bacterial loads, we did not incorporate procedures intended to terminate residual phage activity during sample processing. Residual phage activity during post-treatment processing therefore cannot be excluded. Future studies involving quantitative microbiological endpoints should incorporate validated phage-neutralization procedures to minimize this potential source of bias.

The study was also conducted at a single commercial dairy farm over a limited observation period, which restricts the generalizability of the findings. In addition, the study did not evaluate changes in the broader environmental microbial community. This study did not address reduced phage efficacy within mature biofilms, the potential emergence of phage-resistant bacterial variants, economic comparisons with conventional chemical disinfectants, or regulatory requirements for environmental phage application.

Future multicenter studies should therefore evaluate longer-term efficacy across different management and environmental conditions and incorporate quantitative assessment of bacterial and phage populations. Culture-independent approaches could further characterize changes in microbial community composition and AMR determinants. Additional evaluation of biofilm-associated efficacy, emergence of phage resistance, cost-effectiveness, and regulatory requirements will also be important for establishing evidence-based frameworks for the practical implementation of bacteriophage-based environmental biocontrol in livestock production.

## CONCLUSION

This study demonstrated the feasibility of a farm-adapted multicomponent bacteriophage preparation for environmental biocontrol under commercial dairy cattle housing conditions. Initial screening showed high susceptibility in 42% (16/38) of farm-derived bacterial isolates, whereas adaptive host range expansion increased susceptibility to all 38 tested isolates. The locally isolated *E. coli* phage vB_Eco_BCPEco_001 exhibited lytic activity against most tested *E. coli* strains and was successfully incorporated into the multicomponent preparation. Genomic analysis identified a 170,117-bp genome with 295 genes and revealed no detectable virulence- or AMR-associated genes or integrase-coding sequences. Maltodextrin-based lyophilization resulted in <0.3 log₁₀ PFU/mL loss after reconstitution, and phage viability remained stable for up to 6 months at both 4°C and 25°C (p > 0.05). Following environmental application, several opportunistic pathogenic bacteria detected before treatment were no longer recovered from treated sites, whereas the untreated control area retained bacterial profiles more similar to baseline conditions. No observable adverse effects were detected in calves during or after treatment.

The combination of farm-specific phage isolation, adaptive host range expansion, lyophilization, and environmental application provides a practical framework for tailoring bacteriophage preparations to bacterial populations circulating within individual livestock facilities. Maintaining phage viability during storage at both refrigerated and ambient temperatures may facilitate transportation, storage, and routine on-farm use. Environmental application also offers a potential complementary approach to conventional hygiene and biosecurity practices without direct administration of the preparation to animals.

A major strength of this study was integrating bacterial surveillance, farm-specific phage isolation, adaptive optimization, genomic characterization, formulation development, and field application into a single workflow under routine commercial farm conditions. Nevertheless, the findings were derived from a single farm, qualitative bacterial recovery, and a limited post-treatment observation period. Multicenter studies incorporating quantitative bacterial enumeration, sequential sampling, phage persistence on treated surfaces, culture-independent microbial community analysis, biofilm-associated efficacy, emergence of phage resistance, and economic evaluation are required to establish the durability and generalizability of the observed effects.

Overall, the findings support farm-adapted multicomponent bacteriophage preparations as a promising complementary environmental biocontrol strategy for dairy production systems. The combination of broad lytic coverage, adaptability to locally circulating bacterial strains, formulation stability, and feasibility of application under commercial conditions provides a foundation for further development of phage-based environmental interventions. With validation in larger and longer-term field studies, this approach could contribute to sustainable farm biosecurity and microbial control strategies consistent with One Health principles.

## DATA AVAILABILITY

The data supporting the findings of this study are included in the article and its Supplementary Materials. The genome sequence of *Escherichia* phage vB_Eco_BCPEco_001 is available in GenBank under accession number PV295200. Additional datasets are available from the corresponding authors upon reasonable request.

## GENERATIVE AI DECLARATION

The authors used ChatGPT and OpenAI Codex during the preparation of this manuscript solely for English-language refinement and proofreading. Every AI-assisted suggestion was evaluated, verified, and, where necessary, revised by the authors, who remain fully responsible for the manuscript’s accuracy, originality, and integrity. The AI tool played no role in generating research data, performing statistical analyses, interpreting the findings, or developing scientific conclusions.

## AUTHORS’ CONTRIBUTIONS

ET: Conceptualization, study design, field experiment design, project administration, investigation, bacterio-phage isolation and characterization, host range analysis, EOP and MOI optimization, phage adaptation, preparation of purified phage clones for whole-genome sequencing, formal analysis, data interpretation, and writing–original draft. ND: Farm sampling, sample processing, bacterial isolation, and investigation. SK: Farm sampling, bacteriophage identification and adaptation, novel bacteriophage isolation, and investigation. NT: Bacteriophage identification and adaptation, novel bacteriophage isolation, and investigation. NJ: Development of the lyophilized bacteriophage preparation. GG: Bioinformatic analysis. NP: Coordination of the dairy farm field trial, environmental application of the bacteriophage preparation, and field data collection. MM: Transmission electron microscopy analysis and interpretation. IP: Data analysis and interpretation. RG: Co-supervision, study design, and writing–review and editing. SC: Conceptualization, supervision, study design, data interpretation, and writing–review and editing. All authors read, revised, and approved the final manuscript.
